# Efficacy of topical ruxolitinib for cutaneous dermatomyositis

**DOI:** 10.1016/j.jdcr.2023.09.043

**Published:** 2023-11-21

**Authors:** Aviya Lanis, Hanna Kim, Shajia Lu, Wanxia Li Tsai, Amy Kaneshiro, Alison Ehrlich, George Martin, Adam Schiffenbauer, Susan Shenoi

**Affiliations:** a Department of Pediatric Rheumatology, Seattle Children’s Hospital, University of Washington, Seattle, Washington; b Juvenile Myositis Pathogenesis and Therapeutics Unit, Pediatric Translational Research Branch, National Institute of Arthritis and Musculoskeletal and Skin Diseases, National Institutes of Health, Bethesda, Maryland; c Translational Immunology Section, Office of Science and Technology, National Institute of Arthritis and Musculoskeletal and Skin Diseases, National Institutes of Health, Bethesda, Maryland; d Environmental Autoimmunity Group, Clinical Research Branch, National Institute of Environmental Health Sciences (NIEHS), National Institutes of Health, Bethesda, Maryland; e Dr. George Martin Dermatology Associates, Kihei, Hawaii

**Keywords:** dermatomyositis, pediatric rheumatic disease, ruxolitinib

## Introduction

Dermatomyositis (DM) is a rare systemic inflammatory disorder seen in pediatric and adult patients that primarily affects the skin and muscles, associated with increased interferon (IFN)-regulated proinflammatory cytokines. Oral ruxolitinib, a Janus kinase inhibitor (JAKi), has been used for refractory adult and pediatric DM.^1^, ^2^, ^3^ To date, however, there are no data investigating use of topical ruxolitinib.^3^, ^4^, ^5^ We report the use of topical ruxolitinib for cutaneous manifestations in refractory DM.

## Case 1

A 14-year-old female with P155/140 positive juvenile dermatomyositis diagnosed 7 years ago presented with weakness, violaceous facial rash, and Gottron papules. At diagnosis, she was treated with glucocorticoids (intravenous methylprednisolone and oral prednisone), intravenous immune globulin, hydroxychloroquine, and methotrexate. For refractory disease, she required a multitude of medications in varying combinations, including mycophenolate mofetil, abatacept, and rituximab. She has required daily oral prednisone (0.05-1 mg/kg) and intermittent pulse intravenous methylprednisolone. Complications from steroids include cataract development and steroid myopathy. Currently she receives intravenous tocilizumab, intravenous immune globulin, pulse intravenous methylprednisolone and 0.12 mg/kg daily oral prednisone and has had 6 months of inactive muscle disease.

**Fig 1:**
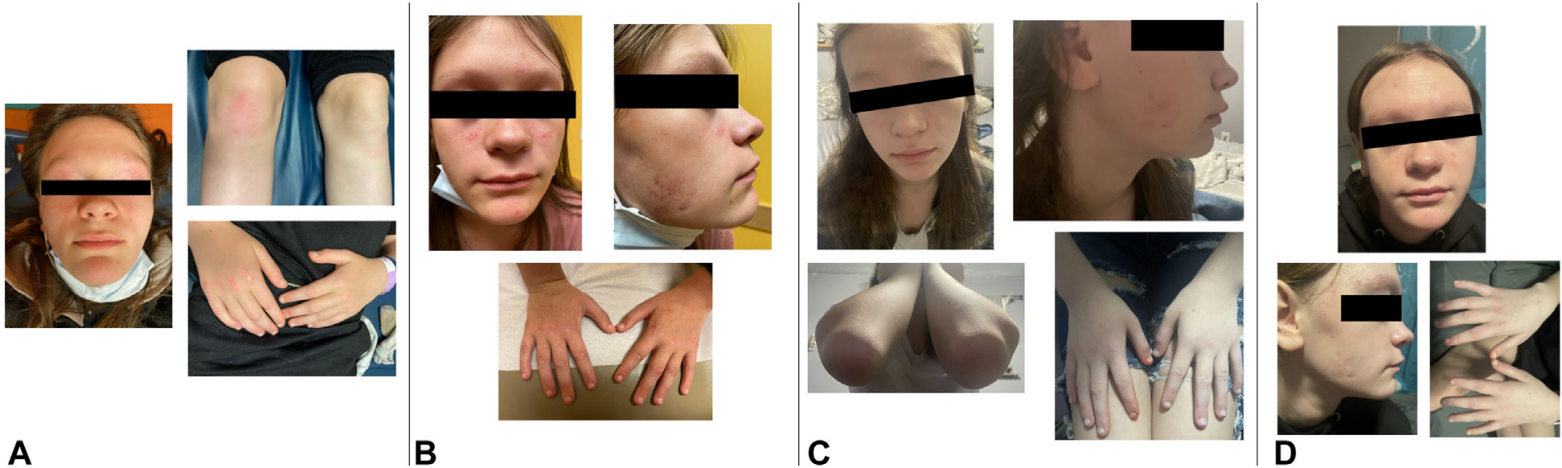
Case 1: **A,** Pre-ruxolitinib rash with ill-defined erythema of upper eyelids and bilateral cheeks with coalescing erythematous flat-topped papules over most extensor metacarpals, proximal and distal interphalangeal joints. **B,** Rash 1 month after initiation of topical ruxolitinib. **C,** Rash 4 months after initiation of topical ruxolitinib. **D,** Rash 7 months after initiation of topical ruxolitinib.

Patient has persistent active cutaneous disease (facial dermatitis, heliotrope rash, Gottron papules, Gottron sign, and facial erythema) (Fig 1, *A*), and abnormal nailfold capillaries. Dermatology recommended desonide 0.05% cream twice daily and pimecrolimus 1% cream twice daily, with transition from pimecrolimus to tacrolimus 0.03% ointment twice daily after 3 months of persistent rash. She reported inconsistent adherence with topicals due to poor efficacy. Four months after initial topical therapy, topical ruxolitinib 1.5% cream twice daily was added. Within 1 month, she had significant improvement in skin disease (Fig 1, *B*) and self-discontinued topical tacrolimus and desonide. Additional quiescent cutaneous disease allowed tapering of oral prednisone from 0.25 mg/kg to 0.12 mg/kg daily, with ongoing improvement at 7 months (Fig 1, *C, D*).

## Case 2

**Fig 2:**
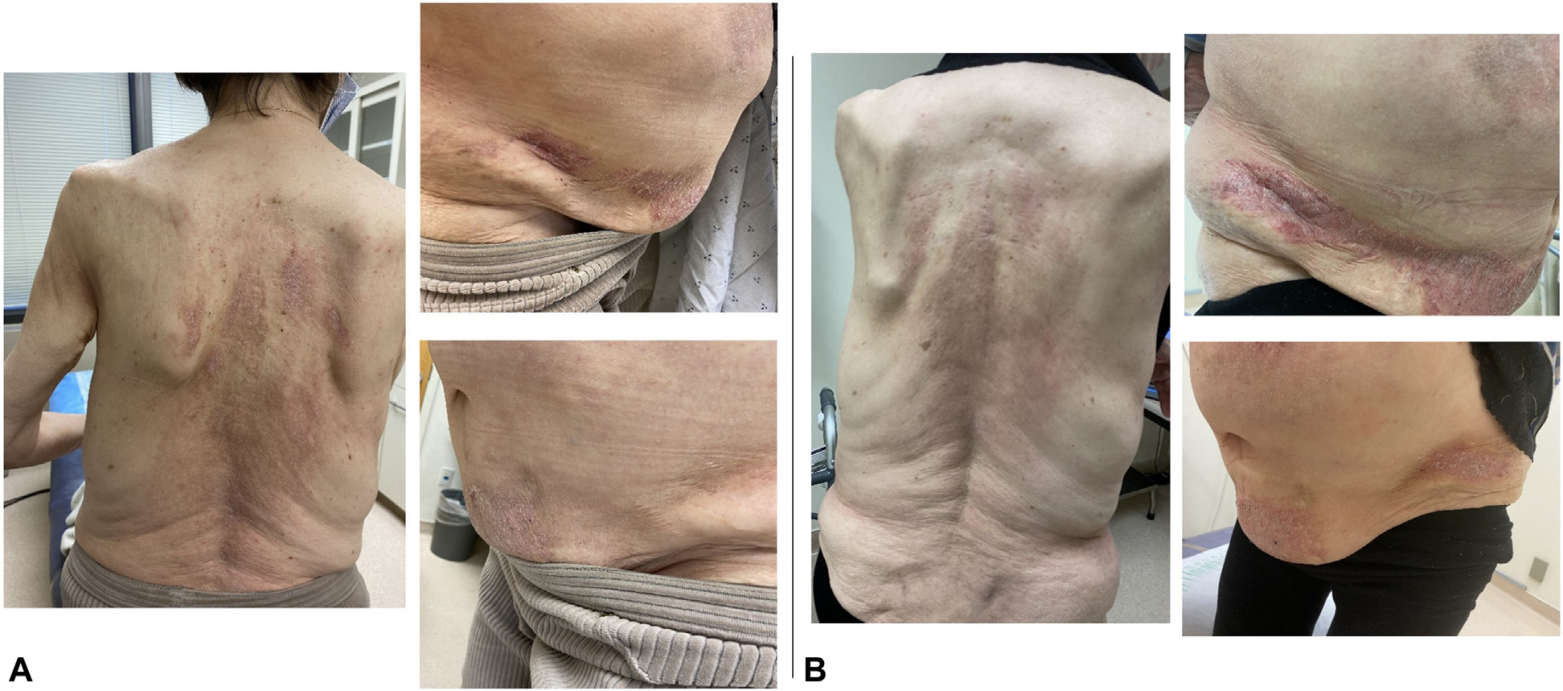
Case 2: **A,** Pre-ruxolitinib rash with erythema of back and abdomen. **B,** Rash 6 months after starting topical ruxolitinib.

**Fig 3:**
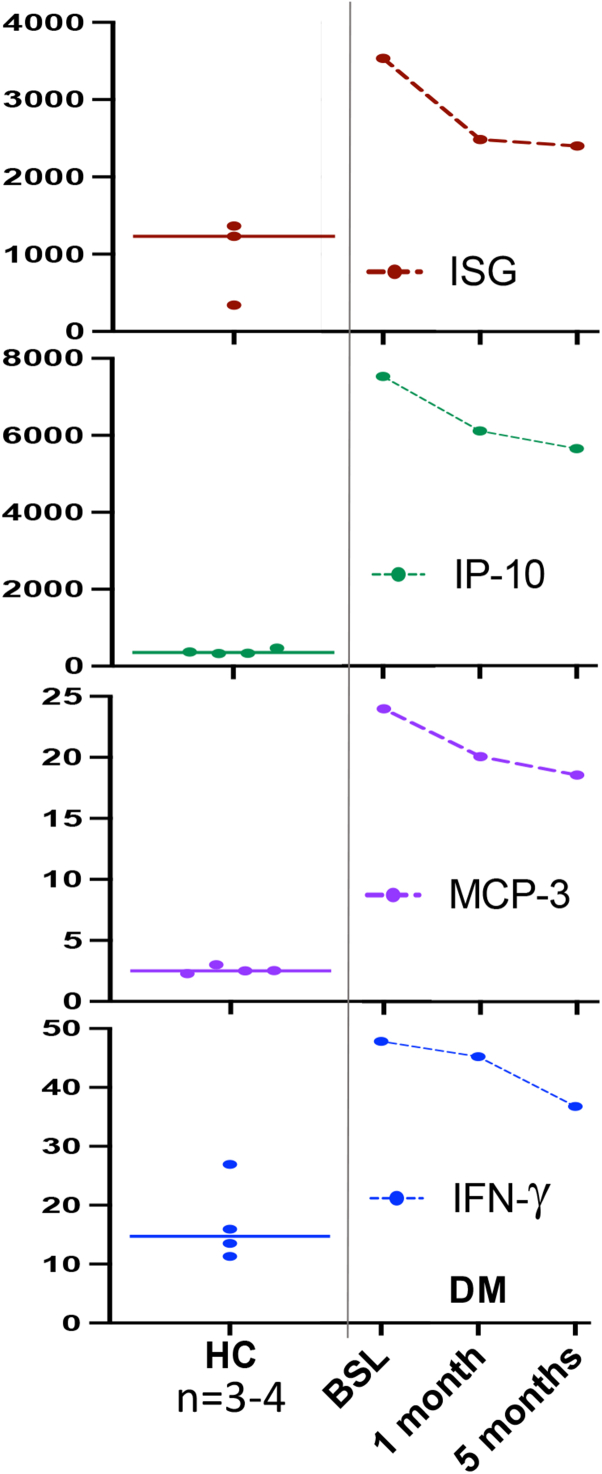
Interferon-stimulated 28-gene score (ISG) and interferon-related proteins (IP-10 or CXCL10, MCP3), and interferon gamma (IFN-γ) top to bottom were measured in 3 to 4 healthy controls (*left*) and case 2 before starting topical ruxolitinib at baseline (BSL) and then 1 and 5 months after (*right*).

A 64-year-old woman was diagnosed 28 years ago with anti-NXP positive DM, presenting with DM rash, muscle weakness and elevated muscle enzymes with confirmatory electromyography and muscle biopsy. Complications included calcinosis with dyslipidemia, anemia, thrombocytopenia, gastroesophageal reflux, diabetes mellitus type 2, and noncirrhotic portal hypertension with varices and portal gastropathy. Treatment in varying combinations included rituximab, intravenous immune globulin, methotrexate, azathioprine, adalimumab, hydroxychloroquine, and cyclosporine. At her most recent visit, she was on 4 mg prednisone daily. Due to persistent rash of abdomen and back, she was started on topical ruxolitinib which was self-discontinued after 6 months of treatment with improvement in rash (Fig 2, *A, B*). Peripheral IFN-related markers (gene and protein) which were elevated before starting topical ruxolitinib and decreased after starting treatment (Fig 3).

## Discussion

Systemic JAKis are promising in several conditions with dermatologic involvement including DM/juvenile dermatomyositis.^1^, ^2^, ^3^, ^4^, ^5^ Topical ruxolitinib is Food and Drug Administration-approved for pediatric vitiligo and atopic dermatitis.^2^ To our knowledge, this is the first case report to demonstrate efficacy of topical ruxolitinib for refractory DM skin disease, allowing corticosteroid taper in case 1. Although these cases were never treated with systemic JAKi, the presumed mechanism of action for both systemic and topical JAKi is targeted inhibition of cytokine-mediated signaling via Janus kinases and Signal Transducers and Activators of Transcription including type I and II IFN.^2^, ^3^, ^4^ Patients with DM and juvenile dermatomyositis have demonstrated elevated peripheral IFN signatures, with case reports showing systemic JAKi can decrease IFN markers and prompt clinical improvement.^2^, ^3^, ^4^ Topical treatment has the benefits of decreased mean plasma concentrations with low incidence of adverse events and stable hematologic markers supporting minimal systemic toxicity.^6^ Side effects of topical ruxolitinib include folliculitis, diarrhea, eosinophilia, headache, viral reactivations, and infections although these were not experienced in our cases. Traditional management of refractory DM skin disease includes increasing systemic medications, but side effect profiles are significant with risk of nonadherence. Topical ruxolitinib should be further studied as an alternative adjunctive therapy and may reduce the need for additional systemic therapy in refractory cutaneous DM.

## Conflicts of interest

Dr Kim is part of a clinical study at NIAMS that received grant support for under a government CRADA from Eli Lilly. Dr Martin is a member of the scientific adviser board for Bristol Meyers Squibb, DUSA/SUN, AbbVie, Ortho/Bausch Health, Galderma, Pfizer, LEO, Celgene, Janssen, Horizon, UCB, Trevi, Almirall, Evelo, and Organogenesis; a speaker for UCB, Almirall, LEO, Incyte, and Dermavant; and a consultant for Bristol Meyers Squibb, DUSA/SUN, AbbVie, Ortho/Bausch Health, Galderma, Pfizer, LEO, Celgene, UCB, Trevi, Almirall, Lilly, Evelo, NobelPharma, and Alumis. Drs Lanis, Ehrlich, and Schiffenbauer and Authors Lu, Tsai, Kaneshiro, and Shenoi have no conflicts of interest to declare.
